# From Facile One-Pot Synthesis of Semi-Degradable Amphiphilic Miktoarm Polymers to Unique Degradation Properties

**DOI:** 10.3390/ma17112684

**Published:** 2024-06-02

**Authors:** Maria Kupczak, Anna Mielańczyk, Tomasz Fronczyk, Patryk Drejka, Przemyslaw Ledwon, Dorota Neugebauer

**Affiliations:** 1 Department of Physical Chemistry and Technology of Polymers, Faculty of Chemistry, Silesian University of Technology, 9. M. Strzody St., 44-100 Gliwice, Poland; maria.kupczak@impib.lukasiewicz.gov.pl (M.K.); tomasz.fronczyk@polsl.pl (T.F.); patryk.drejka@polsl.pl (P.D.); przemyslaw.ledwon@polsl.pl (P.L.); dorota.neugebauer@polsl.pl (D.N.); 2 Łukasiewicz Research Network–Institute for Engineering of Polymer Materials and Dyes, 55. M. Skłodowska-Curie St., 87-100 Toruń, Poland

**Keywords:** *one-pot* synthesis, ATRP, ROP, *click chemistry*, miktopolymers, degradation

## Abstract

We report a *one-pot* synthesis of well-defined A_5_B and A_8_B miktoarm star-shaped polymers where *N,N*-dimethylaminoethyl methacrylate (DMAEMA) and various cyclic esters such as ε-caprolactone (ε-CL), lactide (LA) and glycolide (GA) were used for the synthesis. Miktopolymers were obtained by simultaneously carrying out atom transfer radical polymerization (ATRP) of DMAEMA, ring-opening polymerization (ROP) of cyclic esters, and *click* reaction between the azide group in gluconamide-based (GLBr_5_-Az) or lactonamide-based (GLBr_8_-Az) ATRP initiators and 4-pentyn-1-ol. The relatively low dispersity indices of the obtained miktoarm stars (Đ = 1.2–1.6) indicate that control over the polymerization processes was sustained despite almost complete monomers conversions (83–99%). The presence of salts from phosphate-buffered saline (PBS) in polymer solutions affects the phase transition, increasing cloud point temperatures (T_CP_) values. The critical aggregation concentration (CAC) values increased with a decreasing number of average molecular weights of the hydrophobic fraction. Hydrolytic degradation studies revealed that the highest reduction of molecular weight was observed for polymers with PCL and PLGCL arm. The influence of the composition on the miktopolymers hydrophilicity was investigated via water contact angle (WCA) measurement. Thermogravimetric analysis (TGA) disclosed that the number of arms and their composition in the miktopolymer affects its weight loss under the influence of temperature.

## 1. Introduction

The main goal of *one-pot* synthesis is to improve the efficiency of the chemical reaction by avoiding a long process of separation and purification of intermediate compounds while increasing the yield. More than a hundred years have passed since Sir Robert Robinson performed the first *one-pot* reaction, namely the synthesis of tropinone, and since then, a significant number of *one-pot* strategies have been developed [1,2,3,4,5]. In organic synthesis, one example of a *one-pot* reaction is a multicomponent reaction (MCR). In MCR, at least three starting materials react sequentially with each other in a single reaction vessel, where the order of reaction depends on the chemical affinity of the reagents and formed intermediates [6]. Thanks to this method, Heck and Dömling obtained a 2,4-disubstituted thiazoles complex using oxo components, primary amines, thiocarboxylic acids, and a special isocyanide [7]. Another example is domino reactions (cascade reactions, tandem reactions), which allow the efficient synthesis of complex molecules from relatively simple substrates in a single reaction vessel without the need to purify intermediates [8]. Konieczny et al. obtained 4,7-dihydroxythioaurone derivatives by a *one-pot* reaction of benzaldehydes with 4-acetyl-2-oxo-benz[1,3]oxathioles and piperidine acetate in DMSO [9]. In the case of tandem reactions, variable synthesis conditions can be used [10].

In polymer synthesis, a *one-pot* reaction simultaneously allows for the synthesis and functionalization of polymers and acquires a relatively complex structure in a one-step procedure [11,12,13]. The advantage of this method is the avoidance of a long process of separation and purification of intermediate compounds. Polymers synthesized by this method are usually obtained using various polymerization techniques, such as ring-opening polymerization (ROP) or/and various controlled/“pseudo-living” radical polymerization (CRP) techniques, including atom transfer radical polymerization (ATRP), nitroxide-mediated radical polymerization (NMP) and radical polymerization with reversible fragmentation combined with chain transfer (RAFT) [14,15,16]. The literature describes a few examples of the *one-pot* synthesis of well-defined polymers containing PDMAEMA and aliphatic polyester segments. For example, Mespouille et al. synthesized a well-defined amphiphilic block copolymer PCL-*b*-PDMAEMA with the *one-pot* method by combining ATRP and *click* reactions [17]. Huang et al. obtained star-shaped diblock and triblock copolymers with PCL, PDMAEMA, and/or POEGMA segments via *one-pot* ATRP using bromine end-capped six-arm PCL as a macroinitiator [18]. Zhang et al. obtained a miktoarm star-shaped polymer consisting of PDMAEMA, PCL, and polystyrene (PS) or polyethylene oxide (PEO) arms. They combined techniques such as ROP, ATRP, and *click* reactions [19].

Progress in the development of polymerization methods has led to the possibility of obtaining more complex polymer structures, such as block polymers [20], polymer brushes [21], and star polymers [22], which have completely different properties from their linear analogs. Structures with a star topology include, among others, miktopolymers, which contain three or more arms with different chemical compositions, connected to one central core [23]. Currently, they are obtained using the *coupling-onto*, *in–out*, *arm-first*, and *core-first* methods. Nowadays, designing macromolecules with predetermined properties, and the resulting applications, is desired; hence, the ability to control these properties by varying the polymer arms is essential. Due to their unique properties, which can be adjusted not only by the selection of polymer arms but also by the type of the core, miktoarm polymers are used, among others, in medicine, pharmacy, and biotechnology.

In our previous works concerning the degradation of PDMAEMA/polyester block copolymers, we have shown that enzymatic degradation using Novozyme 435 primarily involved the ester bonds in PDMAEMA side chains, and the rate of polyester degradation decreased with the increase in the chain length of PDMAEMA [24]. Whereas in the case of PDMAEMA/polyester miktoarm stars obtained via an *in–out* approach, enzymatic degradation performed using lipase from *Pseudomonas cepacia* showed an increased degradation rate for the polymer with PCL arms [25].

Our previous research was focused on the synthesis of miktoarm star-shaped polymers via a coupling-onto [26] or in–out method [25]. In this study, a series of well-defined miktoarm star-shaped copolymers based on DMAEMA and cyclic esters were obtained by facile *one-pot* synthesis using a combination of techniques such as ATRP, ROP, and *click* reaction. Miktoinitiators based on the amide derivative of D-gluconic acid containing one azide group and five bromoester groups (GLBr_5_-Az) [26,27], and the amide derivative of *lactobionic acid* containing one azide group and eight bromoester groups (GLBr_8_-Az) have been synthesized and characterized by our group previously [28].

Herein, we present the synthetic pathway to obtain amphiphilic pH- and thermoresponsive miktoarm stars, which additionally are semi-degradable due to the presence of the polyester arm. In this work, compositional properties of the obtained miktoarm stars were evaluated based on their behavior in water solution, WCA measurements on their surface, thermal stability, and hydrolytic degradation rate. It is worth noticing that the one-pot strategy enabled us to obtain polymers with complex structures with high reaction efficiency, and in a relatively short time.

## 2. Materials and Methods

### 2.1. Materials

N,N′-dimethylaminoethyl methacrylate (DMAEMA, Aldrich, St. Louis, MO, USA, 98%) was stored over molecular sieves in a freezer under nitrogen. ε-Caprolactone (CL, Alfa Aesar, 99%, Warsaw, Poland) and toluene were distilled before use and stored over molecular sieves. 4-Pentyn-1-ol (PentAl, Aldrich, 97%), lactide (LA, Aldrich, 99%), N,N,N′,N″,N″-Pentamethyldiethylenetriamine (PMDETA, Aldrich, 99%), glycolide (GA, Aldrich, 99%), and pyrene (Acros, extra dry, 99.5%, Geel, Belgium) were used directly without purification. Copper(I) chloride (CuCl, Fluka, 98%, Steinheim, Germany) was purified by stirring in glacial acetic acid followed by filtration and washing with ethanol and diethyl ether. Subsequently, the solid was dried under a vacuum. Tin(II) bis(2-ethylhexanoate) (Sn (Oct)_2_, Alfa Aesar, 96%) was distilled prior to use. An amount of 0.01 M Phosphate buffer saline (PBS, pH 7.4, Sigma-Aldrich) was prepared according to the following procedure: one tablet was dissolved in 200 mL of deionized water, yielding 0.01 M phosphate-buffered saline (0.0027 M potassium chloride and 0.137 M sodium chloride). Gluconamide-based (GLBr_5_-Az) and lactonamide-based (GLBr_8_-Az) initiators were synthesized earlier [27,28], and methylene chloride (CH_2_Cl_2_) and heptane were used as received.

### 2.2. One-Pot Synthesis of Amphiphilic Miktoarm Star-Shaped Polymers

Monomers (DMAEMA, CL, LA, GA), ligand (PMDETA), ATRP initiator (GLBr_5_-Az, LABr_8_-Az), ROP initiator (PentAl) and toluene were placed in a Schlenk flask (in the case of SMS1 and NMS1 the mixture of reagents in Schlenk flask was degassed by three freeze–pump–thaw cycles; in the case of SMS2-SMS4 and NMS2-NMS4, the reaction mixture was purged with inert gas). After that CuCl and Sn(Oct)_2_ were added, and the reaction flask was immersed in an oil bath at 130 °C. After a predetermined time, the reaction was stopped by exposing the reaction mixture to air. The reaction mixture was diluted with CH_2_Cl_2_, passed through a neutral alumina column to remove the copper catalyst, and concentrated in a rotary evaporator with a vacuum pressure gauge. The polymers were precipitated in n-heptane and dried under vacuum at room temperature to constant mass. Miktopolymers were obtained with yields above 50%. The compositions of the reaction mixtures for the syntheses are given in Tables S1 and S2.

### 2.3. Characterization of Miktoarm Star-Shaped Polymers

In the one-pot polymerization of DMAEMA and CL, the conversions were determined by gas chromatography (GC). The chromatograph (6850 Network GC System, Agilent Technologies, Santa Clara, CA, USA) was equipped with a DB-WAX column 30 m × 0.32 mm × 0.25 µm (Agilent Technologies, Santa Clara, CA, USA) and flame ionization detector. Injector and detector temperatures were kept constant at 250 °C (conditions: anisole as internal standard, column initial temperature 40 °C; column final temperature 200 °C). The conversions were calculated by detecting the decrease in the monomer peak area relative to the standard peak area. In the one-pot polymerization of DMAEMA and LA or GA, the conversions were calculated using ^1^H NMR spectra of the reaction mixtures in CDCl_3_. ^1^H NMR spectra of the polymers were collected on Varian Inova 600 MHz spectrometer (Palo Alto, Santa Clara, CA, USA) at 25 °C using CDCl_3_ as a solvent and TMS as an internal standard. The apparent number-average molecular weights (M_n, SEC_) were determined by size-exclusion chromatograph (SEC, 1100 Agilent 1260 Infinity, Santa Clara, CA, USA,) equipped with an isocratic pump, autosampler, degasser, thermostatic box for columns and differential refractometer MDS RI Detector. Addon Rev. B.01.02 data analysis software (Agilent Technologies, Hongkong, China) was used for data collecting and processing. The SEC calculated molecular weight was based on calibration using linear polystyrene standards (M_p_ = 580–3,000,000 g/mol). Pre-column guard 5 μm 50 × 7.5 mm and double PLGel 5 μm MIXED-C and MIXED-D 300 × 7.5 mm column were used for separation. The measurements were carried out in THF (HPLC-grade) as the solvent at 40 °C with a flow rate of 0.8 mL/min. UV–vis spectroscopy (Evolution 300 Spectrophotometer, Thermo Scientific, Waltham, MA, USA) was used to determine the T_CP_. Optical absorbances of polymers in aqueous solutions (1 mg/mL) were measured at 500 nm at a temperature ranging from 40 to 100 °C with a heating rate of 2 °C/min. T_CP_ was defined as the temperature at which an extremum of the first derivation is located by differentiating the transmittance vs. temperature curve. The critical aggregation concentration (CAC) values of miktoarm polymers water solutions were determined using pyrene as a fluorescence probe. First, the predetermined pyrene solution (5 µM) in ethanol was added to a series of vials, which were further stirred at room temperature in the dark for 24. Next, the miktoarm star-shaped polymers were dissolved in deionized water, and proper solutions within the range 1 × 10^−3^ to 1 mg/mL were added to the vials with pyrene. Final mixtures were stirred in the dark for 24 h before measurement The fluorescence emission spectra (λ_ex_ = 337 nm) of polymer/pyrene solutions were measured on a fluoroSENS Pro-11 spectrofluorimeter (CAMLIN, Lisburn, Ireland). The CAC value was defined as the point of intersection of two lines in the plot of intensity ratio (I_1_/I_3_) from pyrene emission spectra vs. the logarithm of the polymer concentration (logC, where C is concentration in mg/mL). The water contact angle (WCA) was determined using the sessile drop method utilizing the OCA 15EC (Data Physics, Filderstadt, Germany) goniometer. Drops (4 µL) of deionized water were placed on the polymer surfaces using an end-flat micrometric syringe (B.Braun Inject-F, luer solo). Water droplets were dropped onto at least three different sites of each lozenge surface. Dynamic results were recorded, and the average value of the contact angle was obtained for each sample. The experimental error was ±2°. WCA was determined using the image analysis provided with the SCA20 software and calculated with the Owens, Wendt, Rabel, and Kaelble (OWRK) method. The polymeric samples were in the form of lozenges with a diameter equal to 12 mm and a height of 1 mm, which were obtained by pressing the polymer in a press with a pressure of 10 tons. Thermogravimetric analysis (TGA) was accomplished using a TGA 8000 thermogravimetric analyzer (PerkinElmer, Rodgau, Germany) at a heating rate of 10 °C/min over a temperature range from 30 to 600 °C under a nitrogen flow.

### 2.4. Hydrolytic Degradation

First, approximately 5 ± 0.25 mg of each polymeric sample was weighed in glass vials. Next, 5 mL of PBS solution at pH 7.4 or 5.0, with the addition of sodium azide (23 µmoles of NaN_3_ per 5 mL of PBS), was added. The vials and their contents were sealed and placed on Orbital Shaker-Incubator ES-80 set to 150 rpm at a temperature of 37 ± 2 °C. Samples were taken out of the incubator at predetermined time intervals, i.e., 1, 2, 4, 6, and 9 weeks, then frozen and lyophilized. Samples were analyzed in triplicate.

## 3. Results and Discussion

### 3.1. One-Pot Synthesis of Miktoarm Stars

**Scheme 1:**
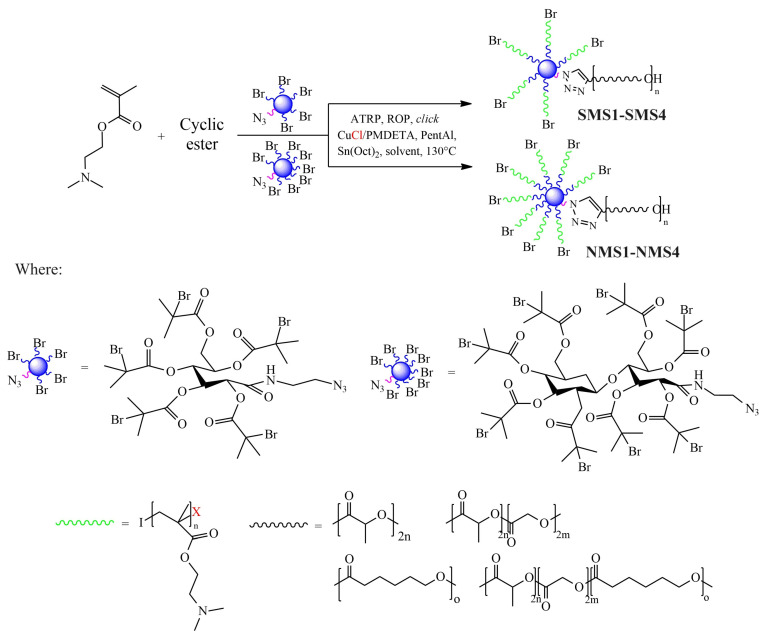
Schematic representation for the synthesis of amphiphilic star-shaped miktoarm polymers.

Well-defined A_5_B-type six-armed miktoarm star-shaped (SMS) polymers and A_8_B-type nine-armed miktoarm star-shaped (NMS) polymers were obtained through *one-pot* synthesis combining ATRP, ROP and *click* reaction (Scheme 1). Although there was a difference in the number of initiating bromoester groups (5 vs. 8), in all cases, after two hours, the conversions of monomers were in the range of 83–99%.

**Figure 1:**
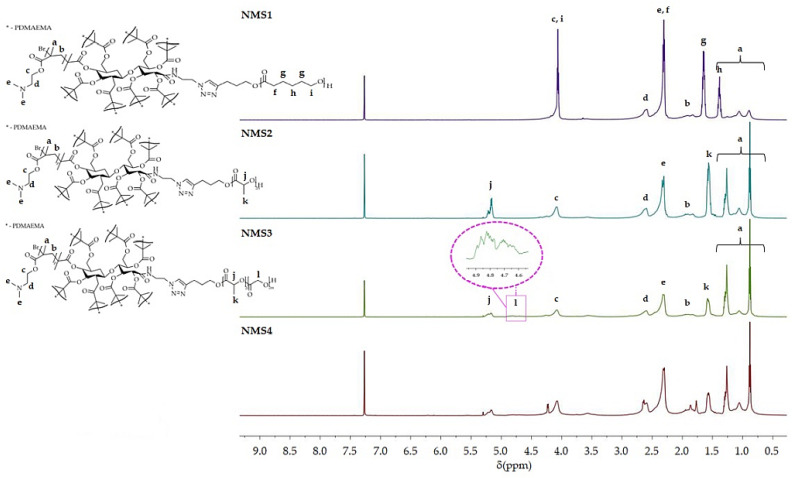
^1^H NMR (600 MHz, CDCl_3_) spectra of nine-arm star-shaped miktopolymers.

The characterization of the obtained six-arm star-shaped (SMS1-SMS4) and nine-arm star-shaped (NMS1-NMS4) copolymers are presented in Table 1. The structures of polymers were confirmed by ^1^H NMR analysis. ^1^H NMR spectra of the reaction mixtures were used to calculate the degree of polymerization (DP) of cyclic (di)esters in PLA, PLGA, and PCLGA, while GC analysis was used to calculate the DP of PCL. Figure 1 presents ^1^H NMR spectra of miktopolymers with characteristic peaks belonging to PDMAEMA and a proper polyester segment. Each spectrum contained signals from PDMAEMA, i.e., protons from the main chain (methyl groups at the range from 0.5 to 1.2 ppm, and methylene groups at the range from 1.8 to 2.0 ppm) and protons belonging to the pendant groups (two broad singlets from -N(CH_3_)_2_ at 2.2 ppm, and -CH_2_- near to carbonyl group at 2.5 ppm). In addition, at 3.65 ppm, there were protons from the methylene group close to the amine. In the case of copolymers with PLA, protons from methylidyne and methyl groups were observed at 5.2 ppm and 1.55 ppm, respectively. PCL gives four characteristic signals, i.e., a multiplet from methylene groups in the main chain at δ = 1.65 ppm and δ = 1.4 ppm, a triplet from the methylene group linked to the carbonyl group at δ = 2.31 ppm, and a triplet from the methylene group bonded to oxygen at δ = 4.06 ppm. However, a triplet from the methylene group bonded to the hydroxyl group at the chain-end at the shift δ = 3.66 ppm. The signal of the triazole protons overlaps with the signal from the deuterated solvent.

**Figure 2:**
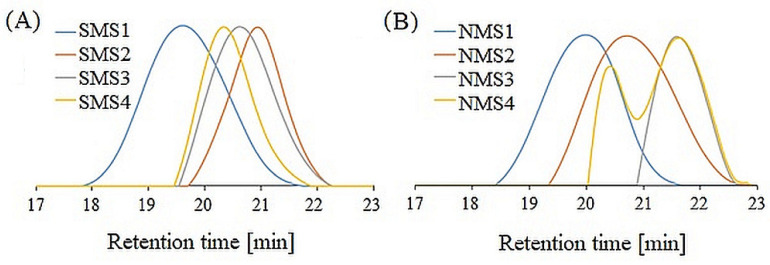
SEC traces of six-arm (**A**) and nine-arm miktopolymers (**B**).

The SEC traces of miktoarm star-shaped copolymers are shown in Figure 2. Rather low dispersity values (Ð) (1.1–1.5) for the majority of samples indicate that the control over the polymerization processes was sustained. The exception is nine-arm NMS4 with terpolyester (PLGCL) arm, whose SEC eluogram shows a bimodal curve, which indicates star–star coupling as a result of the recombination of polymethacrylate arms. Presumably, the coupling reactions occurred due to high arm density and high monomer conversion [29].

The number-average molecular weights determined using a refractive index detector (M_n; SEC_) for six-arm miktopolymers ranged from 4800 to 14,200 g/mol and were lower than theoretical ones (M_n; the_ = 25,000–27,000 g/mol). A similar dependence was found for nine-arm miktopolymers, where M_n; SEC_ ranged from 3000 to 11,900 g/mol and corresponding M_n; theo_ = 24,000–28,000 g/mol. The differences in the hydrodynamic volumes of the tested samples and the PS standard result from both the chemical nature (PDMAEMA/polyester samples are amphiphilic and PS is hydrophobic) and topology (star-shaped vs. linear) were determined. Moreover, the molecular structures with five or eight short PDMAEMA arms and one long polyester arm probably influenced their radius of gyration [30], causing the shift toward higher retention time values, as was observed in our previous studies [26].

### 3.2. Miktoarm Stars Behavior in Water Solution

The behavior of obtained miktoarm star-shaped polymers was investigated in water and PBS (pH 7.4) solutions, depending on their composition and architecture, by determination of the cloud point temperature (T_CP_) of polymeric solutions by UV-vis (Table 1). The first derivative method was used to receive the T_CP_ value.

**Figure 3:**
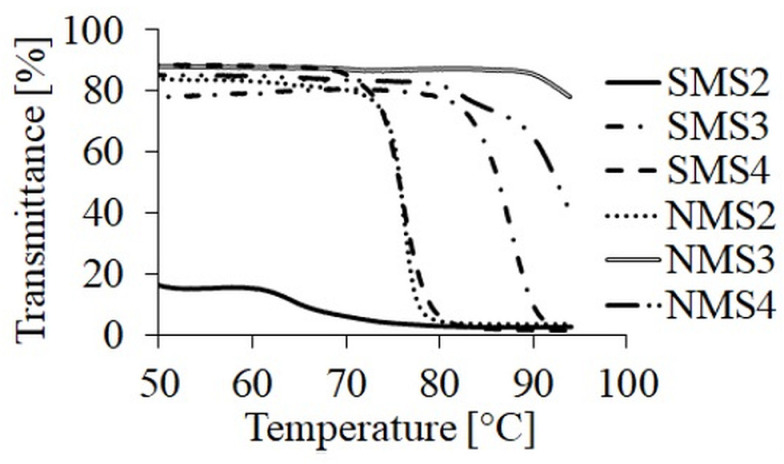
T_CP_ curves of miktopolymers in PBS at pH 7.4.

Polymer samples dissolved in demineralized water did not show a visible and sharp phase transition point as opposed to the same samples dissolved in 0.01 M PBS. These results are in a good agreement with our previous studies [26]. Salts contained in PBS may cause an increase in ionic strength, leading to the replacement of water molecules with dissociated salt ions, which may lead to the appearance of the phase transition point of the tested polymer solutions [31]. The dependences of T_CP_ on polymer composition and topology are shown in Figure 3. The presence in the structure of hydrophobic groups had a significant impact on whether the sample showed characteristic T_CP_. In the case of miktoarm polymers solutions in PBS, the introduction of the GA repeating units into the polyester arm increased the T_CP_ value. The comparison of polymers with the same composition but a different number of arms let us to a conclusion that the additional three PDMAEAM chains increased the stability of a random coil structure in the solution by suppressing the occurrence of hydrophobic interaction between polymeric chains.

**Figure 4:**
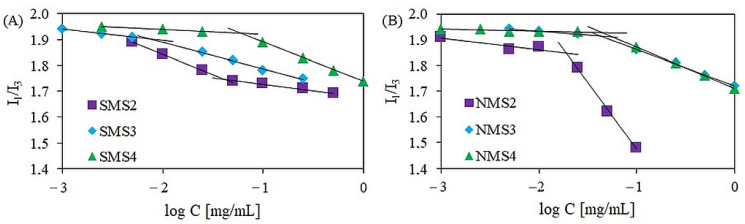
The plot of intensity versus log C for six-arm (**A**) and nine-arm (**B**) miktopolymers.

The critical aggregation concentration (CAC) values were monitored by fluorimetry in the presence of pyrene as a hydrophobic fluorescent probe. The CAC values of amphiphilic polymers were related to the ratio of hydrophilic blocks to hydrophobic ones. As shown in Figure 4, the ratio of I_1_ to I_3_ varied in response to different copolymer concentrations. The CAC values of amphiphilic six-arm and nine-arm star-shaped miktopolymers increased with increasing hydrophilic fraction content. The lowest CAC values were obtained for polymers in which the number-average molecular weight of the polyester arm was the highest. These results are consistent with those obtained earlier by our group for six-armed A5B type miktoarm polymers of the same composition but with different arm lengths, synthesized using the coupling-onto method [26].

### 3.3. Thermal Stability

**Figure 5:**
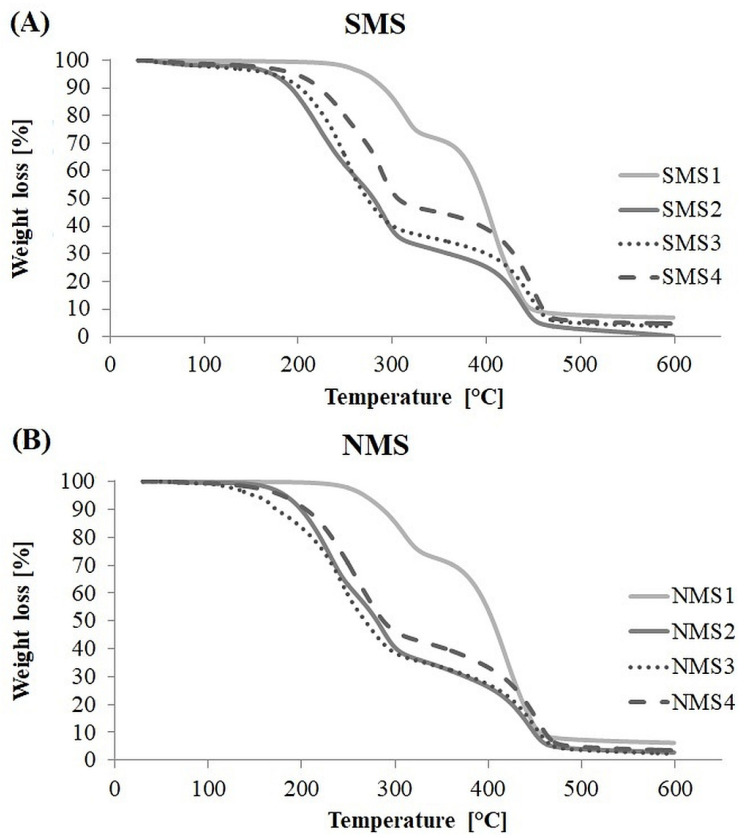
TGA curves of six-arm (**A**) and nine-arm (**B**) miktopolymers.

The data from the TGA analysis are shown in Figure 5. In most cases, the TGA curves showed three decomposition stages at temperature range 200–350 °C and 350–500 °C, attributed to the presence of PDMAEMA and polyester arm in the polymer structure (Figure S1). The first stage indicates thermal decomposition of PDMAEMA side groups, whereas in the second stage, destruction of the PDMAEMA main chain overlaps with the thermal decomposition of a polyester arm [32,33,34,35]. Miktoarm polymers with a PCL arm, that is, SMS1 and NMS1, showed considerable thermal resistance in comparison to all tested samples. SMS1 and NMS1 were stable to the same temperature equal 267 °C, and for both the first derivative peak temperature (T_p_) for the first TGA slope was ca. 310 °C, and the second ca. 410–418 °C. Miktopolymers with a PLA arm, that is, SMS2 and NMS2, showed the most visible three decomposition stages with T_p1_ equal to 223 °C (SMS2) or 232 °C (NMS2), T_p2_ equal to 295 °C (SMS2) and 287 °C (NMS2), and T_p3_ equal to 437 °C or 444 °C. In the case of miktopolymers with a PLGA and PLGCL arm in the structure, the decomposition of nine-arm miktopolymers samples started at temperature of ca. twenty degrees lower (NMS3: 149 °C and NMS4: 179 °C) than for six-arm analogs (SMS3:174 °C SMS4: 198 °C). Since NMS have shorter PDMAEMA arms than SMS, this indicates that the shorter the PDMAEMA arm, the faster weight loss begun. At the same time, the final sample weight loss occurred at slightly higher temperatures for nine-arm miktopolymers than for six-arm analogs with a PCL or PLA arm, indicating that miktopolymers with greater number of PDMAEMA arms are less prone to decomposition under temperature increase in an inert atmosphere. The resistance of the sample to the weight loss also depended on the composition of the polyester arm, and the decrease was as follows: PCL > PLGCL > PLGA > PLA.

### 3.4. Wettability and Hydrophilicity

**Figure 6:**
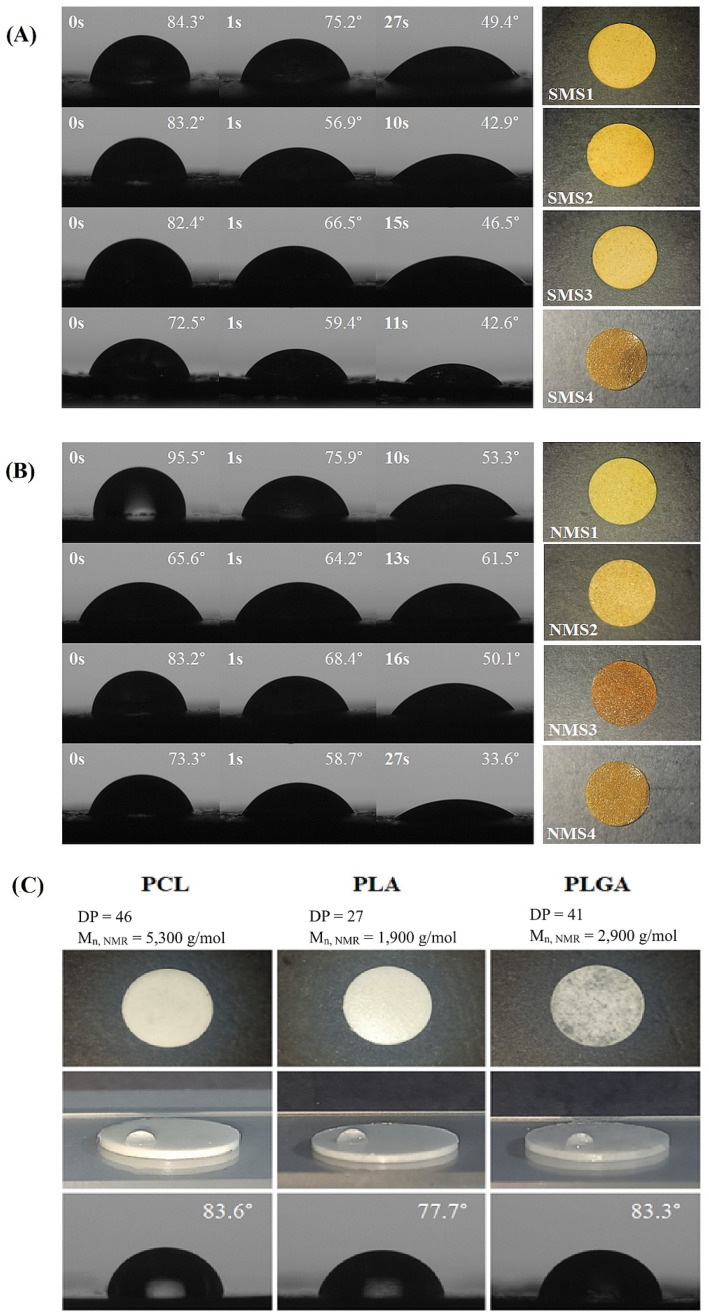
WCA images and photos of six-arm (**A**) and nine-arm (**B**) miktopolymers as well as PCL, PLA, and PLGA (**C**) in the form of lozenges.

The difference in the hydrophilicity of miktopolymers, which impacts the hydrolytic degradation course, was evaluated by water contact angle (WCA) measurements. Additionally, WCA measurements on PCL, PLA and PLGA lozenges were performed, and the obtained contact angle values corresponded to literature data [36,37,38]. As can be seen in Figure 6, values of WCA obtained for miktoarm polymers decreased with the measurement time. This indicates that due to the increased affinity of PDMAEMA chains to the water, the dynamic reorganization of the macromolecules occurred at the interface between the sample surface and water. All tested samples eventually showed stable values of contact angle in the range 33.6° < WCA < 61.5°, indicating that despite the presence of a relatively long hydrophobic polyester arm, the sample in a solid state retained its hydrophilic character. The surface of the NMS2 lozenge appeared to be the most stable, with the angle decreasing by 4° compared to the samples of the other miktopolymers. Samples with PCL arms, despite having the highest molar fraction of DMAEMA, displayed the highest values of WCA. Although both miktopolymers had the same PCL chain length, the PDMAEMA arms of NMS1 were shorter than SMS1. In contrast, SMS4 and NMS4 with PLGCL arms with the lowest DP_CL_, DP_LA_ and DP_GA_ values, in comparison with polyester arms of other miktopolymers, showed the lowest WCA values. These results demonstrate that both the composition and the chain length can be used in order to tailor the surface hydrophylicity.

### 3.5. Hydrolytic Degradation

**Figure 7:**
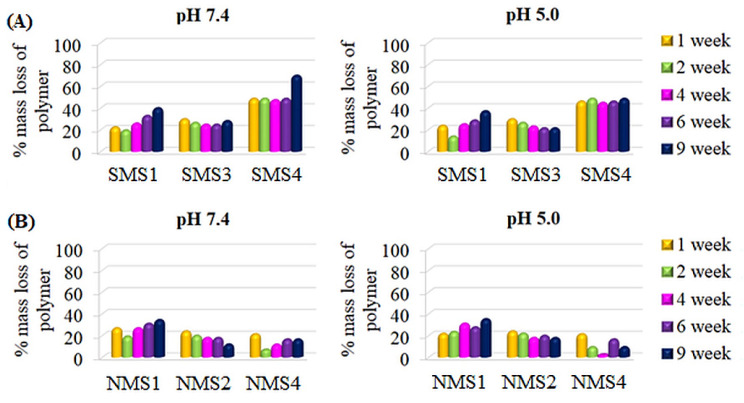
Percentage M_n,SEC_ loss of A_5_B (**A**) and A_8_B (**B**) type miktopolymers.

In the case of the A_5_B-type miktopolymers, the degradation rate decreased as follows: PDMAEMA_5_PLGCL (SMS4) > PDMAEMA_5_PCL (SMS1) > PDMAEMA_5_PLGA (SMS3) (Figure 7A). The comparison of SMS3 (F_DMAEMA_ = 0.32; M_n, theo_ = 25,200 g/mol) with SMS4 (F_DMAEMA_ = 0.41; M_n, theo_ = 24,000 g/mol) led us to a conclusion that the polymer with lower theoretical molecular weight and higher content of hydrophilic fraction degraded faster. Moreover, comparing SMS1 (F_DMAEMA_ = 0.49; M_n, theo_ = 26,100 g/mol) with SMS3, miktopolymers with higher molecular weight and higher values of F_DMAEMA_ degraded slower than SMS3 until 4 weeks. This indicates that at the beginning of the hydrolysis process, the water uptake and ester bond cleavage were faster in macromolecules with lower molecular weights. However, ultimately, macromolecules with the higher content of hydrophilic fraction reached higher values of percentage molar mass loss after 9 weeks.

**Figure 8:**
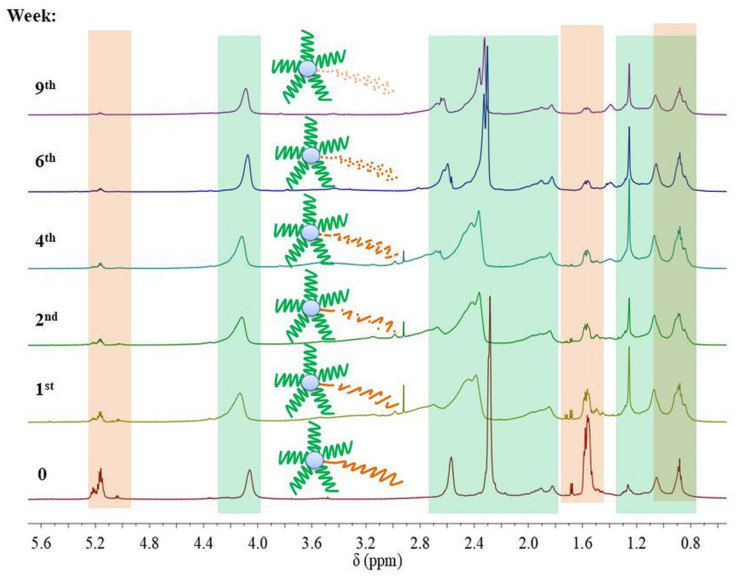
^1^H NMR spectra (600 MHz, CDCl_3_) for SMS2 and its samples after degradation in PBS at pH 7.4, where the green area indicates signals from PDMAEMA arms and the orange area indicates signals from the polyester part. Chemical shifts and integration values of signals assigned to SMS2 are presented in Table S3.

Slightly different behavior was observed in the case of the nine-arm compounds. The degradation course of the NMS1 sample, containing a PCL arm with the highest hydrophilic content (F_DMAEMA_ = 0.49, M_n, theo_ = 26,200 g/mol) but higher theoretical molecular weight than NMS4 (F_DMAEMA_ = 0.41, M_n, theo_ = 24,200 g/mol), showed the fastest molecular weight reduction. Contrastingly, NMS4, with the PLGCL arm, showed the slowest molecular weight reduction (Figure 7B), which could be a result of the imperfect molecular structure of the macromolecule caused by star–star coupling during NMS4 synthesis manifested as a bimodal signal on the SEC chromatogram (Figure 2B). The formation of large macromolecules, which consist of several stars linked by the common PDMAEMA arms, could slow down the water diffusion inside the particle aggregates created by amphiphilic macromolecules in water and be followed by a longer time of polyester degradation entrapped inside the particle aggregate. Moreover, for SMS2 and NMS3, an initial increase in M_n, SEC_ value was observed, and then a slight decrease (Figure S2). However, the intensity of the RID signal corresponding to the polymeric sample gradually decreased with the degradation time. First, we concluded that this follows a gradual increase in PDMAEMA content in polymeric samples caused by polyester degradation and enhanced interactions of polymer with the column, or hydrolysis of (2-hydroxyethyl)dimethylamine groups leading to the formation of methacrylic acid repeating units. However, ^1^H NMR analysis excluded the hydrolysis of amine groups from polymethacrylate arms at pH 7.4, because the ratio of the intensity of protons within methyl groups bonded to amine, to the intensity of signals corresponding to the protons in methyl groups belonging to polymethacrylate backbone was not changed. It was, however, observed that signals of all protons shifted to higher values of δ, which can be seen in Figure 8. This could be caused by the small amount of salts that were still present in samples after degradation. In addition, it was observed that despite the increase in molecular weights determined by SEC analysis, the proton signals in the polyester chain in ^1^H NMR spectra were decreasing, indicating that the degradation of the polyester arm progressed over time.

**Figure 9:**
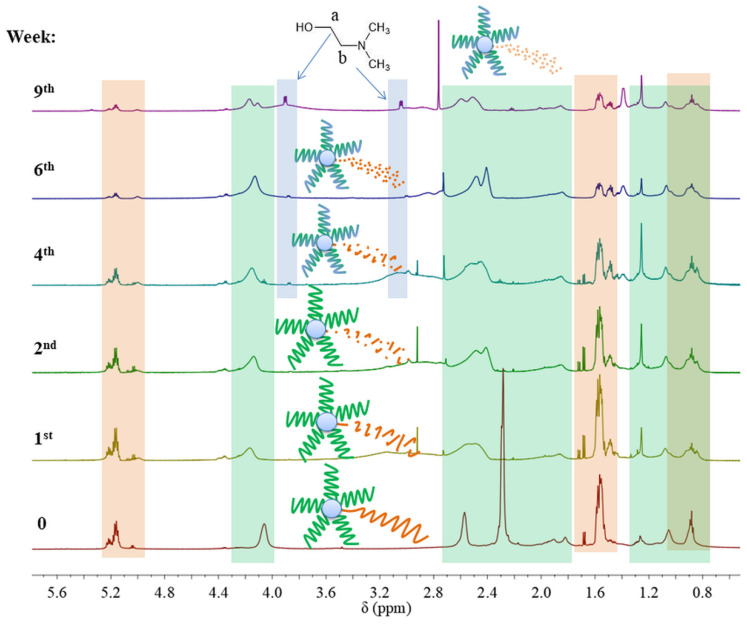
^1^H NMR spectra (600 MHz, CDCl_3_) for SMS2 and its samples after degradation in PBS at pH 5.0 where the green area indicates signals from PDMAEMA arms and the orange area indicates signals from the polyester part. Chemical shifts and integration values of signals assigned to SMS2 are presented in Table S3.

However, ^1^H NMR spectra of samples SMS2-SMS4 and NMS2-NMS4 that were subjected to a pH 5.0 revealed that the ratio of signal intensities of protons from methyl groups in the amine to intensities of protons from methyl groups in the main chain changed. This could indicate that the hydrolysis of DMAEMA occurred only in miktopolymers with LA repeating units in the polyester arm. In addition, the signals of protons from groups linked to the nitrogen atom began to separate. Moreover, at about the fourth week of degradation, two new triplets appeared at 3.08 and 3.90 ppm, indicating the presence of *N, N*-dimethylethanolamine (DMAE) formed during the hydrolysis of DMAEMA-pending groups (Figure 9). The proton signals from the PLA arm decreased gradually in SMS2 and NMS2 samples, whereas signals from PLGCL (SMS4 and NMS4) and from PLGA (SMS3 and NMS3) disappeared completely after one or two weeks, respectively (Figure S3–S9).

**Figure 10:**
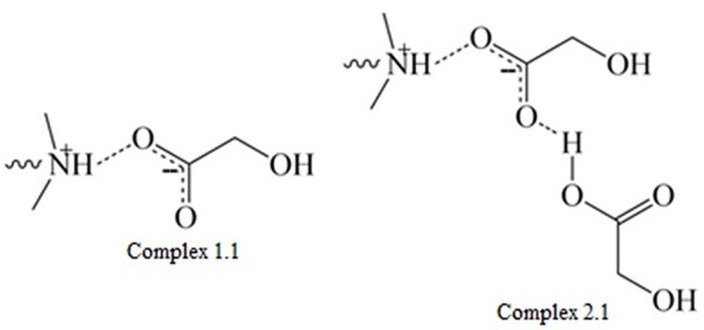
The interaction of the acids formed during degradation with a tertiary amine in the polymethacrylate chain.

In the case of miktoarm polymers with a PLGA or PLGCL arm, a broad signal ranging from 4.0 to 3.5 ppm was observed and then shifted towards higher ppm values. We assume that the shift of this broad signal was the result of ester formation due to reaction between glycolic acid and DMAE. Due to the high susceptibility and speed of degradation of chain fragments composed of GA repeating units, a large fraction of glycolic acid was formed in the first two to four weeks, which then could react with DMAE formed at a later stage—the signal at 3.5 ppm disappeared and appeared at around 4.0 ppm. The signal in the range from 4.7 to 4.0 ppm belongs to PLA, PLGA, or PLGCL oligoesters. The signal shift toward the upfield can be attributed to the degradation of these oligomers and the formation of corresponding acids. Moreover, taking into account the values of the dissociation constants for hydroxyl acids formed during hydrolysis of esters, it can be concluded that glycolic acid (K_a_ = 1.48 × 10^−4^) and lactic acid (K_a_ = 1.41 × 10^−4^) are stronger acids than caproic acid (K_a_ = 1.4 × 10^−5^). This indicates the interaction of the acids formed during the degradation with the tertiary amine in the polymethacrylate side chain, forming complexes (1.1) and (2.1) (Figure 10).

Complex (1.1) is formed by the interaction of one acid molecule with an amine, and complex (2.1) results from the interaction of another acid molecule with the already-formed complex (1.1). The formation of these complexes is controlled by various mechanisms. Complexation of (1.1) is controlled by the acid–base ion evaporation reaction and is highly dependent on the pKa. The complexation of (2.1), on the other hand, occurs through the formation of hydrogen bonds, which should not be directly dependent on pKa [39,40]. Thus, the observed increase in molecular weight values determined by SEC, despite the hydrolysis of the polyester chains, is most likely due to interactions between the tertiary amine groups present in the polymethacrylate arms and the hydroxy acids formed during hydrolysis.

## 4. Conclusions

The use of a *one-pot* synthesis approach allowed the preparation of a wide range of amphiphilic miktoarm polymers that respond to temperature stimuli in water and PBS within the temperature range that excludes these polymers from drug delivery system applications. The CAC values of miktoarm polymers depend on the content of hydrophobic segments and are in the range of 0.008–0.060 mg/mL. TGA analysis revealed that the number of arms and their composition affected the values of temperatures at which the weight loss occurred and the most stable miktoarm polymers are those with a PCL arm. Miktoarm polymers in a solid state demonstrated dynamic surface changes on exposure to water contact. However, the final stable values of WCA indicate the hydrophilic character of all samples and were the lowest for miktoarm polymers with PLGCL arms. The hydrolytic degradation results indicate that the presence of arms with amine functional groups in the structure of miktoarm polymers facilitates the process of polyester degradation and increases the spectrum of potential applications of the obtained polymers. The obtained amphiphilic miktopolymers, due to the presence of biodegradable polyester arm and thermo-/pH-sensitive polymethacrylate arms, can be used in medicine, agriculture, or cosmetology.

## Figures and Tables

**Table 1:** Characterization of miktoarm stars with six (SMS) or nine (NMS) arms obtained via *one-pot* synthesis.

No	DP_DMAEMA_ (x [%])	DP_ε-CL_ (x [%])	DP_LA_ (x [%])	DP_GA_ (x [%])	F_DMAEMA_	M_n, theo_ [g/mol]	M_n, SEC_ [g/mol]	Đ	T_CP, H_2_O_ [°C]	T_CP, PBS_ [°C]	CAC [mg/mL]
SMS1	94 (94)	99 (99)	-	-	0.49	26,100	14,200	1.50	n. d.	n. d.	n. d.
SMS2	88 (88)	-	162 (81)	-	0.35	25,500	4800	1.27	58.4	63.6	0.040
SMS3	83 (83)	-	129 (86)	50 (100)	0.32	25,200	6000	1.35	-	87.8	0.008
SMS4	88 (88)	34 (67)	72 (90)	19 (95)	0.41	24,000	8000	1.23	-	75.5	0.060
NMS1	95 (95)	99 (99)	-	-	0.49	26,200	11,900	1.39	n. d.	n. d.	n. d.
NMS2	86 (86)	-	192 (96)	-	0.31	27,400	5000	1.48	66.7	76.6	0.019
NMS3	92 (92)	-	141 (94)	50 (99)	0.33	27,500	3000	1.36	-	>90.0	0.042
NMS4	89 (89)	33 (66)	74 (92)	19 (97)	0.41	24,200	9900/2600 ^a^	1.55 ^b^	-	>90.0	0.045

Where: SMS1 and NMS1: [DMAEMA]_0_:[ε-CL]_0_:[I]_0_:[PentAl]_0_:[PMDETA]_0_:[CuCl]_0_:[Sn(Oct)_2_]_0_ = 100:100:1:1:1:1:1; 9% weight toluene versus monomer; SMS2 and NMS2: [DMAEMA]_0_:[LA]_0_:[I]_0_:[PentAl]_0_:[PMDETA]_0_:[CuCl]_0_:[Sn(Oct)_2_]_0_ = 100:100:1:1:1:1:1; 9% weight toluene versus monomer; SMS3 and NMS3: [DMAEMA]_0_:[LA]_0_:[GA]_0_:[I]_0_:[PentAl]_0_:[PMDETA]_0_:[CuCl]_0_:[Sn(Oct)_2_]_0_ = 100:75:25:1:1:1:1:0.1; 9% weight toluene versus monomer; SMS4 and NMS4: [DMAEMA]_0_:[ε-CL]_0_:[LA]_0_:[GA]_0_:[I]_0_:[PentAl]_0_:[PMDETA]_0_:[CuCl]_0_:[Sn(Oct)_2_]_0_ = 100:50:40:10:1:1:1:1:0.01; 9% weight toluene versus monomer; x—monomer conversion; DP—degree of polymerization calculated based on GC and ^1^H NMR analyses. F_DMAEMA_—a molar fraction of DMAEMA repeating units in miktopolymer; Đ—dispersity; a—Mp values of the first and second signal at chromatogram; b—the total dispersity value; n. d.—not dissolved; -—soluble in water but no T_CP_ was observed.

## Data Availability

The data presented in this study are available on request from the corresponding author.

## Supplementary Materials

The following supporting information can be downloaded at: https://www.mdpi.com/article/10.3390/ma17112684/s1, Table S1. The compositions of the reaction mixtures for the performed syntheses of A_5_B type miktoarm star-shaped polymers. Table S2. The compositions of the reaction mixtures for the performed syntheses of A_8_B type miktoarm star-shaped polymers. Figure S1. The thermogravimetric analysis (TGA) and derivative thermogravimetric (DTG) curves of the miktoarm polymers in a nitrogen atmosphere. Figure S2. Plots of M_n,SEC_ versus the degradation time for SMS2 and NMS3. Figure S3. ^1^H NMR (600 MHz, CDCl3) spectra of SMS1 with assigned chemical shifts and integration values of signals at pH 7.4 (A), and pH 5.0 (B). Figure S4. ^1^H NMR (600 MHz, CDCl3) spectra of NMS1 with assigned chemical shifts and integration values of signals at pH 7.4 (A), and pH 5.0 (B). Figure S5. ^1^H NMR (600 MHz, CDCl3) spectra of NMS2 with assigned chemical shifts and integration values of signals at pH 7.4 (A), and pH 5.0 (B). Figure S6. ^1^H NMR (600 MHz, CDCl3) spectra of SMS3 with assigned chemical shifts and integration values of signals at pH 7.4 (A), and pH 5.0 (B). Figure S7. ^1^H NMR (600 MHz, CDCl3) spectra of NMS3 with assigned chemical shifts and integration values of signals at pH 7.4 (A), and pH 5.0 (B). Figure S8. ^1^H NMR (600 MHz, CDCl3) spectra of SMS4 with assigned chemical shifts and integration values of signals at pH 7.4 (A), and pH 5.0 (B). Figure S9. ^1^H NMR (600 MHz, CDCl3) spectra of NMS4 with assigned chemical shifts and integration values of signals at pH 7.4 (A), and pH 5.0 (B). Table S3. Chemical shifts and integration values of signals assigned to SMS2 at pH 7.4, and pH 5.0.
