# Supplementary material for: From Facile One-Pot Synthesis of Semi-Degradable Amphiphilic Miktoarm Polymers to Unique Degradation Properties
Source: Materials (Basel). 2024 Jun 2;17(11):2684. doi: 10.3390/ma17112684 (PMC11173590; doi:10.3390/ma17112684)
Supplement: Supplementary file 1 [file materials-17-02684-s001.zip › materials-2986465-supplementary.pdf]

From Facile *One-pot* Synthesis of Semi-degradable Amphiphilic Miktoarm Polymers to Unique Degradation Properties

Maria Kupczak[1,2], Anna Mielańczyk[1]*, Tomasz Fronczyk[1], Patryk Drejka[1], Przemyslaw Ledwon[1], Dorota Neugebauer[1]

[1]*Department of Physical Chemistry and Technology of Polymers, Faculty of Chemistry, Silesian University of Technology, 9. M. Strzody St,, 44-100 Gliwice, Poland*

[2]*Łukasiewicz Research Network – Institute for Engineering of Polymer Materials and Dyes, 55. M. Skłodowska-Curie St., 87-100 Toruń, Poland*

Corresponding author: anna.mielanczyk@polsl.pl

Table of content

1. Composition of the reaction mixtures SMS1-SMS4 and NMS1-NMS4 2
2. TGA thermograms and 1st Derivative 3
3. Hydrolytic degradation – $M_{n,SEC}$ vs the degradation time for SMS2 and NMS3 4
4. Hydrolytic degradation – representative ^{1}HNMR spectra of SMS1-SMS4 and NMS1-NMS4 samples taken during experiment 4

1. Composition of the reaction mixtures SMS1-SMS4 and NMS1-NMS4

Table S1. The compositions of the reaction mixtures for the performed syntheses of A_5B type miktoarm star-shaped polymers

	DMAEMA	CL/LA/GA	$GLBr_5$-Az	CuCl	PMDETA	PentAl	$Sn(Oct)_2$	Toluene
SMS1								
Molar ratio	100	100/-/-	1	1	1	1	1	-
V [ml]	2.000	1.300	-	-	0.025	0.011	0.038	0.330
m [g]	1.866	-	0.11963	0.01175	0.02057	0.00998	0.04809	-
SMS2								
Molar ratio	100	-/100/-	1	1	1	1	0,1	-
V [ml]	2.000	-	-	-	0.025	0.011	0.004	0.37
m [g]	1.866	-/1.71075/-	0.11963	0.01175	0.02057	0.00998	0.00481	-
SMS3								
Molar ratio	100	-/75/25	1	1	1	1	0.01	-
V [ml]	2.000	-	-	-	0.025	0.011	0.0004	0.36
m [g]	1.866	-/1.28306/0.34442	0.11963	0.01175	0.02057	0.00998	0.00048	-
SMS4								
Molar ratio	100	50/40/10	1	1	1	1	0.01	-
V [ml]	2.000	0.7/-/-	-	-	0.025	0.011	0.0004	0.35
m [g]	1.866	-/ 0.68430/0.13777	0.11963	0.01175	0.02057	0.00998	0.00048	-

Table S2. The compositions of the reaction mixtures for the performed syntheses of A_8B type miktoarm star-shaped polymers

	DMAEMA	CL/LA/GA	$LABr_8$-Az	CuCl	PMDETA	PentAl	$Sn(Oct)_2$	Toluene
NMS1								
Molar ratio	100	100/-/-	1	1	1	1	1	-
V [ml]	2.000	1.300	-	-	0.025	0.011	0.038	0.33
m [g]	1.866	-	0.19208	0.01175	0.02057	0.00998	0.04809	-
NMS2								
Molar ratio	100	-/100/-	1	1	1	1	0.1	-
V [ml]	2.000	-	-	-	0.025	0.011	0.004	0.37
m [g]	1.866	-/1.71075/-	0.19208	0.01175	0.02057	0.00998	0.00481	-
NMS3								
Molar ratio	100	-/75/25	1	1	1	1	0.01	-
V [ml]	2.000	-	-	-	0.025	0.011	0.0004	0.36
m [g]	1.866	-/1.28306/0.34442	0.19208	0.01175	0.02057	0.00998	0.00048	-
NMS4								
Molar ratio	100	50/40/10	1	1	1	1	0.01	-
V [ml]	2.000	0.700	-	-	0.025	0.011	0.0004	0.35
m [g]	1.866	-/0.68430/0.13777	0.19208	0.01175	0.02057	0.00998	0.00048	-

2. TGA thermograms and 1st Derivative

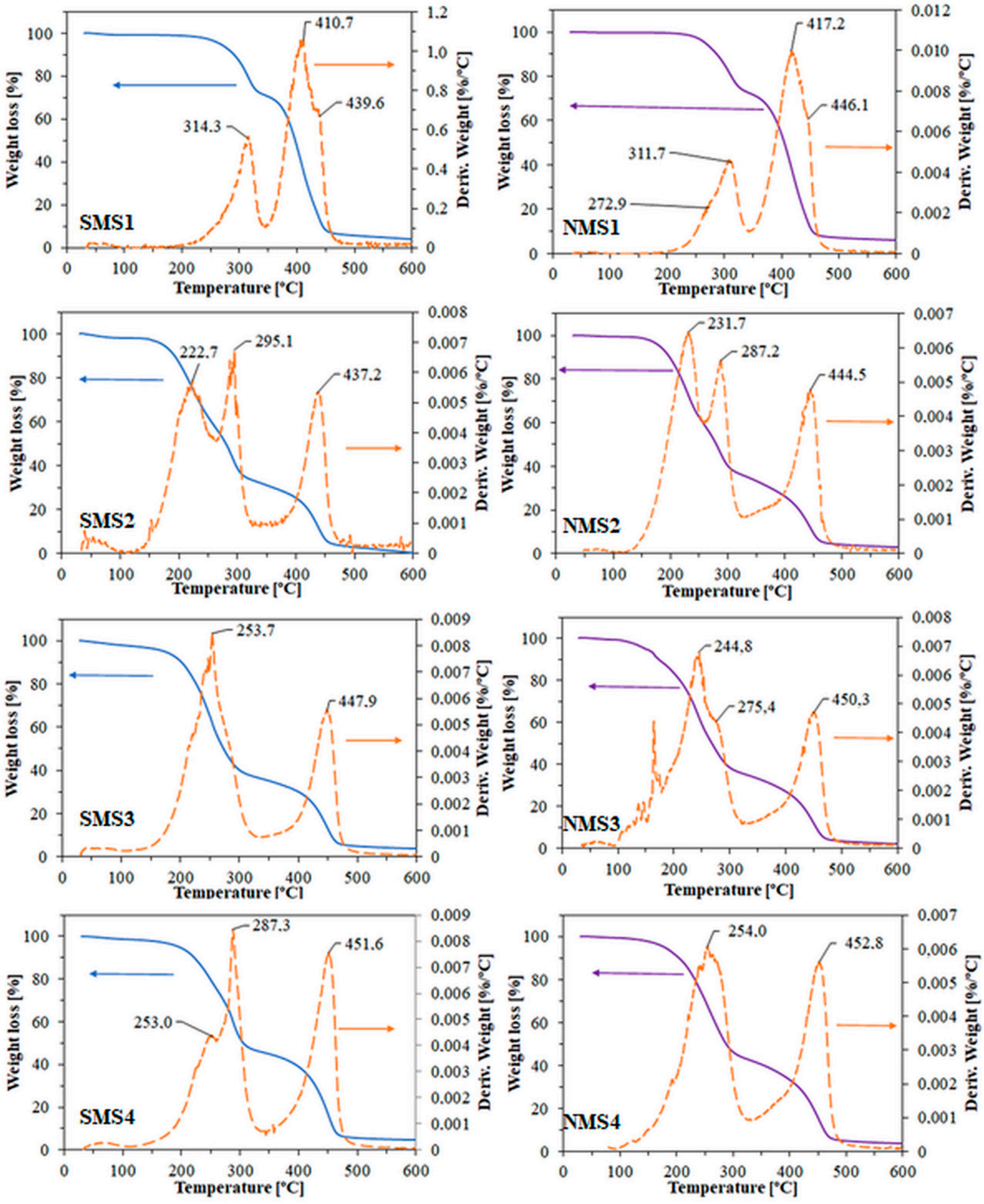

Figure S1. The thermogravimetric analysis (TGA) and derivative thermogravimetric (DTG) curves of the miktoarm polymers in a nitrogen atmosphere.

3. **Hydrolytic degradation – $M_{n,SEC}$ vs the degradation time for SMS2 and NMS3**

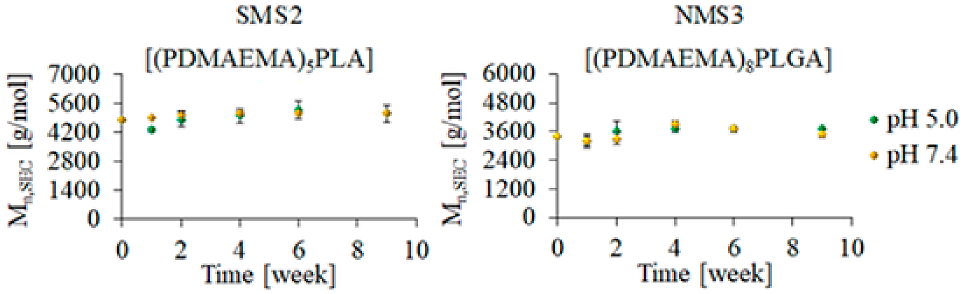

Figure S2. Plots of $M_{n,SEC}$ versus the degradation time for SMS2 and NMS3.

4. **Hydrolytic degradation – representative ^{1}HNMR spectra of SMS1-SMS4 and NMS1-NMS4 samples taken during experiment**

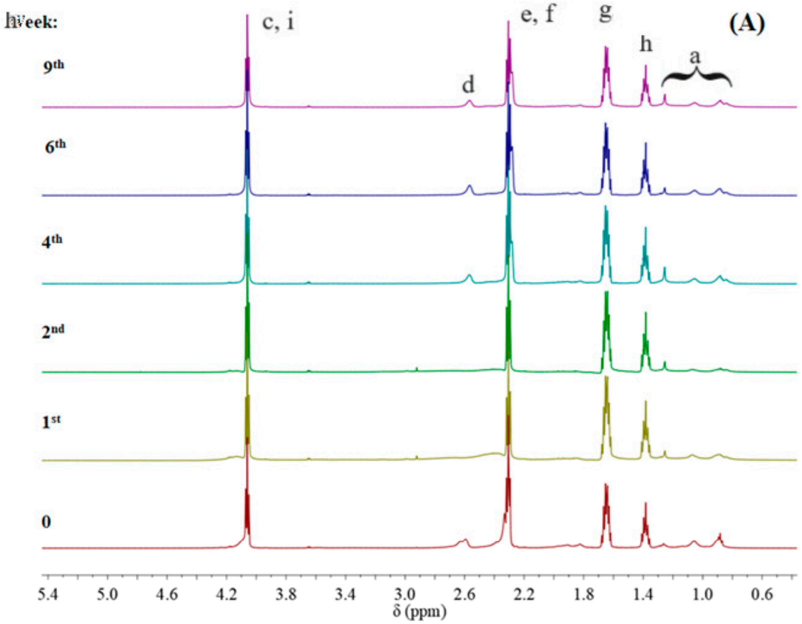

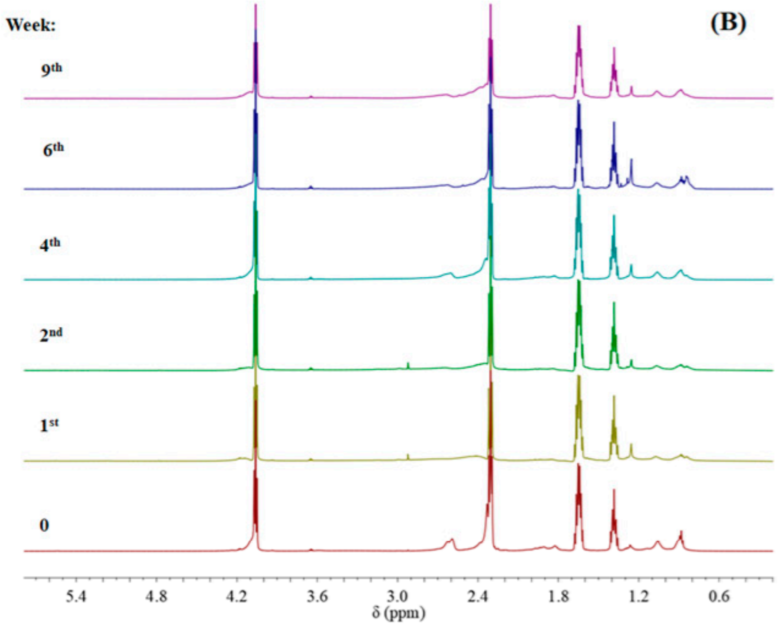

SMS1 pH 7.4	week 0		week 9th	
Signal	Chemical shift (ppm)	Integration value (normalized)	Chemical shift (ppm)	Integration value (normalized)
C*H2*$_{(i)}$O (t, 2H); C*H2*$_{(c)}$O (m, 2H)	4.29 - 3.87	1.00	4.29 - 3.87	1.00
NC*H2*$_{(d)}$ (m, 2H)	2.85 - 2.52	0.29	2.85 - 2.52	0.29
N(C*H3*)$_{(2e)}$ (m, 6H), C*H2*$_{(f)}$C(O)O (t, 2H)	2.46 - 2.18	1.65	2.46 - 2.18	1.65
C*H2*$_{(b)}$ (m, 2H)	2.09 - 1.74	0.29	2.09 - 1.74	0.29
C*H2*$_{(g)}$ (m, 4H)	1.73 - 1.52	1.54	1.73 - 1.52	1.54
C*H2*$_{(h)}$ (m, 2H)	1.44 - 1.31	0.81	1.44 - 1.31	0.81
C*H3*$_{(a)}$ (m, 3H)	1.31 - 0.71	1.09	1.31 - 0.71	1.09

SMS1 pH 50	week 0		week 9th	
Signal	Chemical shift (ppm)	Integration value (normalized)	Chemical shift (ppm)	Integration value (normalized)
C*H2*$_{(i)}$O (t, 2H); C*H2*$_{(c)}$O (m, 2H)	4.36 - 3.87	1.00	4.36 - 3.87	1.00
NC*H2*$_{(d)}$ (m, 2H)	2.98 - 2.54	0.49	2.98 - 2.54	0.46
N(C*H3*)$_{(2e)}$ (m, 6H), C*H2*$_{(f)}$C(O)O (t, 2H)	2.54 - 2.15	1.91	2.54 - 2.15	1.70
C*H2*$_{(b)}$ (m, 2H)	2.08 - 1.75	0.35	2.08 - 1.75	0.30
C*H2*$_{(g)}$ (m, 4H)	1.75 - 1.56	1.14	1.75 - 1.56	1.30
C*H2*$_{(h)}$ (m, 2H)	1.45 - 1.32	0.59	1.45 - 1.32	0.70
C*H3*$_{(a)}$ (m, 3H)	1.31 - 0.65	0.88	1.31 - 0.65	1.05

Figure S3. ^{1}H NMR (600 MHz, $CDCl_3$) spectra of SMS1 with assigned chemical shifts and integration values of signals at pH 7.4 (A), and pH 5.0 (B).

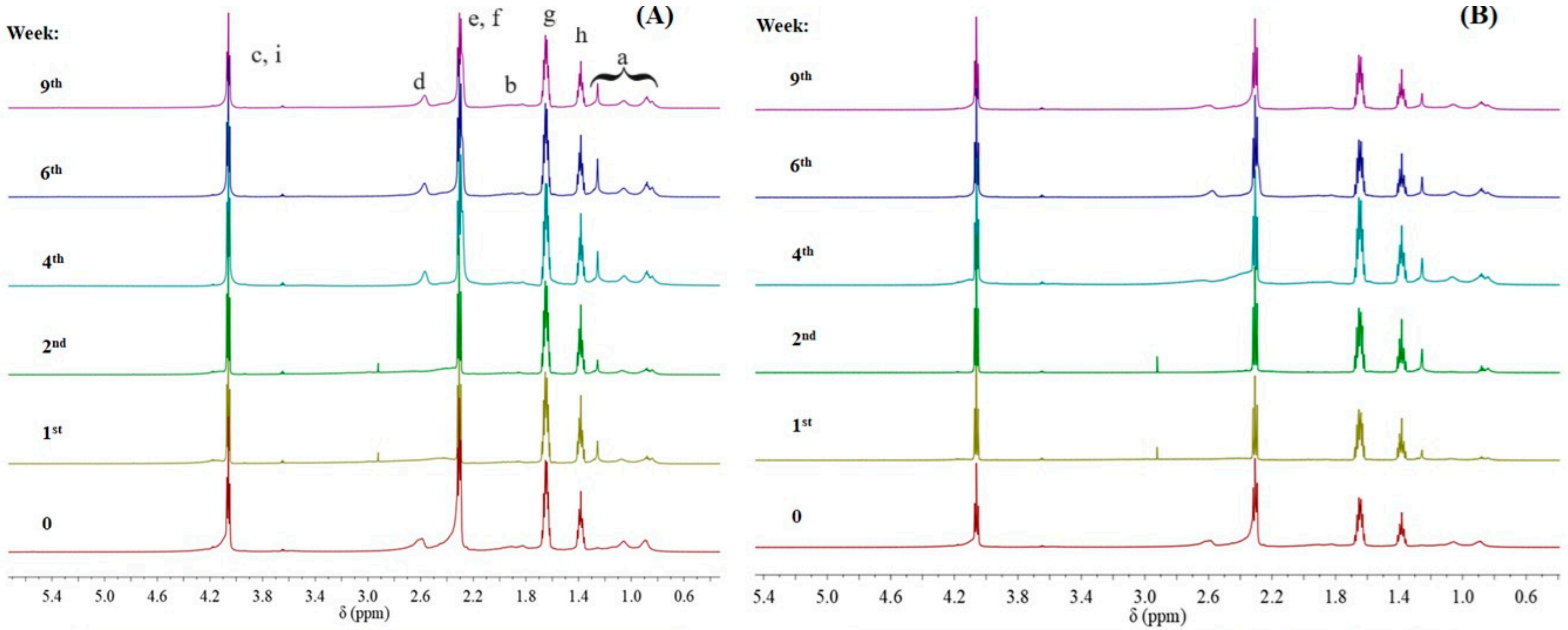

NMS1 pH 7.4	week 0		week 9th	
Signal	Chemical shift (ppm)	Integration value (normalized)	Chemical shift (ppm)	Integration value (normalized)
$CH2_{(i)}$O (t, 2H); $CH2_{(c)}$O (m, 2H)	4.23 - 3.94	1.00	4.23 - 3.94	1.00
N$CH2_{(d)}$ (m, 2H)	2.71 - 2.46	0.63	2.71 - 2.46	0.60
N($CH3$)$_{(2e)}$ (m, 6H), $CH2_{(f)}$C(O)O (t, 2H)	2.46 - 2.14	1.96	2.46 - 2.14	1.95
$CH2_{(b)}$ (m, 2H)	2.14 - 1.73	0.55	2.14 - 1.73	0.47
$CH2_{(g)}$ (m, 4H), $CH2_{(h)}$ (m, 2H)	1.73 - 1.31	1.89	1.73 - 1.31	2.07
$CH3_{(a)}$ (m, 3H)	1.31 - 0.65	0.96	1.31 - 0.65	1.24

NMS1 pH 5.0	week 0		week 9th	
Signal	Chemical shift (ppm)	Integration value (normalized)	Chemical shift (ppm)	Integration value (normalized)
$CH2_{(i)}$O (t, 2H); $CH2_{(c)}$O (m, 2H)	4.24 - 3.93	1.00	4.24 - 3.93	1.00
N$CH2_{(d)}$ (m, 2H)	2.78 - 2.44	0.75	2.78 - 2.44	0.59
N($CH3$)$_{(2e)}$ (m, 6H), $CH2_{(f)}$C(O)O (t, 2H)	2.44 - 2.18	1.84	2.44 - 2.18	1.71
$CH2_{(b)}$ (m, 2H)	2.07 - 1.71	0.52	2.07 - 1.71	0.38
$CH2_{(g)}$ (m, 4H), $CH2_{(h)}$ (m, 2H)	1.71 - 1.33	1.82	1.71 - 1.33	2.10
$CH3_{(a)}$ (m, 3H)	1.33 - 0.59	0.98	1.33 - 0.59	1.46

Figure S4. ^{1}H NMR (600 MHz, $CDCl_3$) spectra of NMS1 with assigned chemical shifts and integration values of signals at pH 7.4 (A), and pH 5.0 (B).

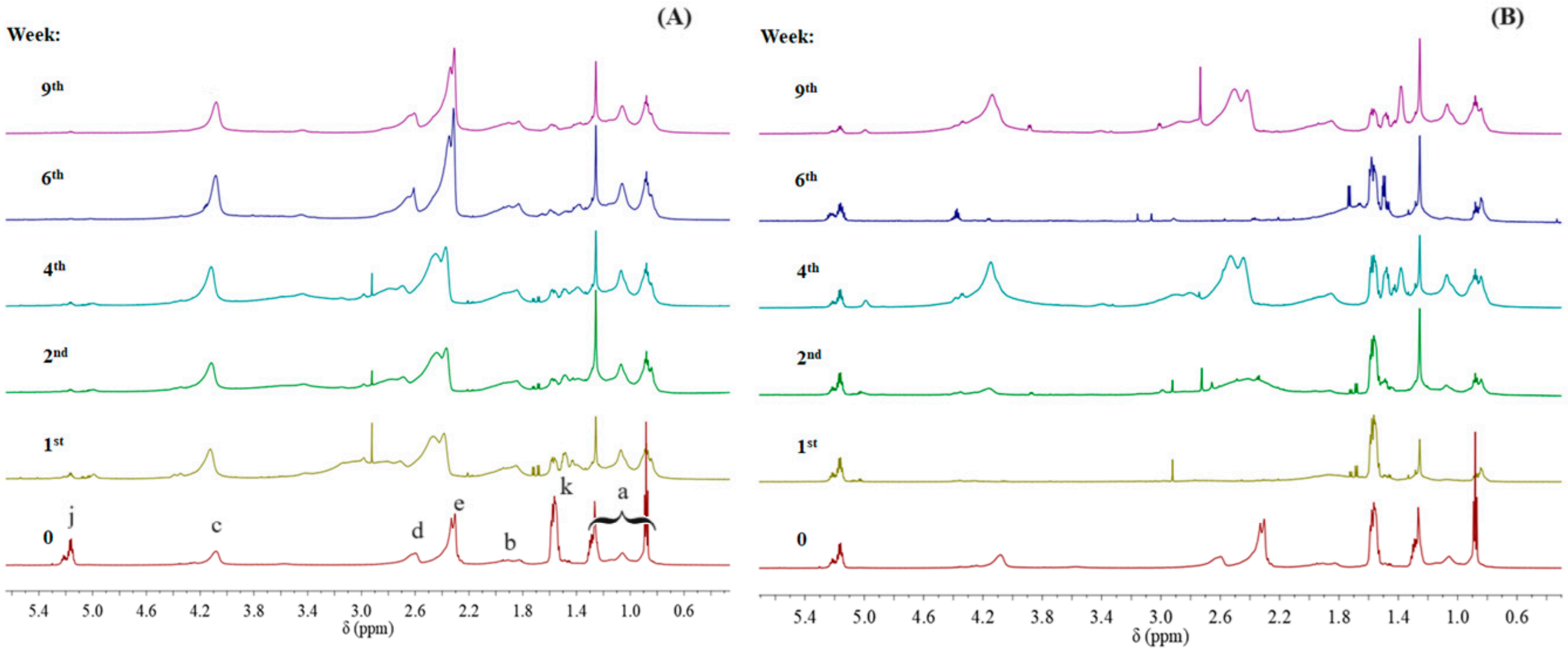

NMS2 pH 7.4	week 0		week 9th	
Signal	Chemical shift (ppm)	Integration value (normalized)	Chemical shift (ppm)	Integration value (normalized)
C*H*(j)O (m, 1H)	5.31 - 5.09	1.00	5.31 - 5.09	0.09
C*H2*(c)O (m, 2H)	4.42 - 3.88	1.50	4.42 - 3.88	1.69
N*CH2*(d) (m, 2H), N(C*H3*)(2e) (m, 6H)	3.24 - 2.22	5.50	3.24 - 2.22	5.22
C*H2*(b) (m, 2H)	2.20 - 1.73	1.19	2.20 - 1.73	1.34
C*H3*(k) (m, 3H)	1.73 - 1.35	3.58	1.73 - 1.35	1.00
C*H3*(a) (m, 3H)	1.35 - 0.67	5.58	1.35 - 0.67	4.02

NMS2 pH 5.0	week 0		week 9th	
Signal	Chemical shift (ppm)	Integration value (normalized)	Chemical shift (ppm)	Integration value (normalized)
C*H*(j)O (m, 1H)	5.35 - 5.07	1.00	5.35 - 5.07	1.00
C*H2*(c)O (m, 2H)	4.44 - 3.95	1.45	4.44 - 3.95	15.55
N*CH2*(d) (m, 2H), N(C*H3*)(2e) (m, 6H)	3.17 - 2.24	5.40	3.17 - 2.24	28.32
C*H2*(b) (m, 2H)	2.22 - 1.72	1.21	2.22 - 1.72	6.60
C*H3*(k) (m, 3H)	1.72 - 1.32	3.61	1.72 - 1.32	11.21
C*H3*(a) (m, 3H)	1.32 - 0.64	5.46	1.32 - 0.64	21.23

Figure S5. ^{1}H NMR (600 MHz, $CDCl_3$) spectra of NMS2 with assigned chemical shifts and integration values of signals at pH 7.4 (A), and pH 5.0 (B).

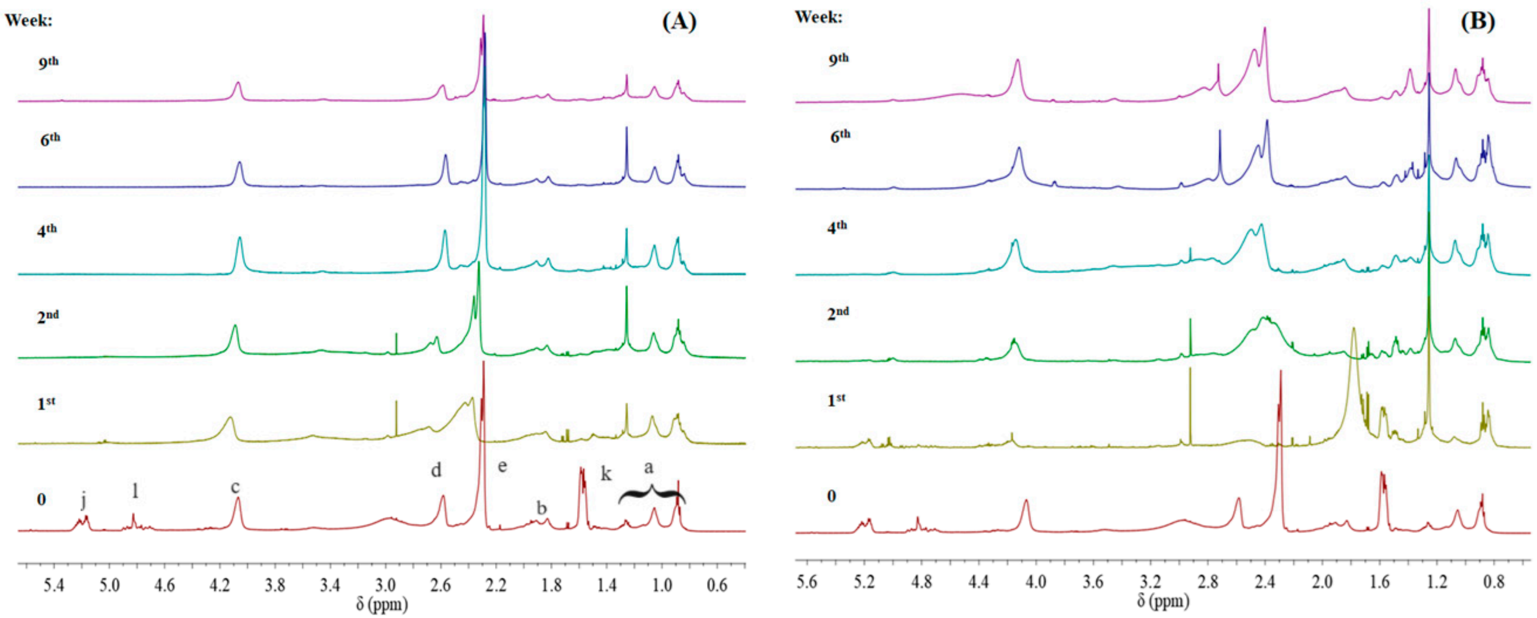

SMS3 pH 7.4	week 0		week 9th	
**Signal**	**Chemical shift (ppm)**	**Integration value (normalized)**	**Chemical shift (ppm)**	**Integration value (normalized)**
$CH_{(j)}O$ (m, 1H)	5.36 - 5.02	1.00	5.36 - 5.02	1.00
$CH2_{(l)}O$ (m, 2H)	4.95 - 4.54	1.04	4.95 - 4.54	1.17
$CH2_{(c)}O$ (m, 2H)	4.33 - 3.86	2.07	4.33 - 3.86	4.08
$NCH2_{(d)}$ (m, 2H), $N(CH3)_{(2e)}$ (m, 6H)	3.22 - 2.15	9.36	3.22 - 2.15	13.22
$CH2_{(b)}$ (m, 2H)	2.09 - 1.65	1.80	2.09 - 1.65	3.39
$CH3_{(k)}$ (m, 3H)	1.65 - 1.32	2.47	1.65 - 1.32	2.21
$CH3_{(a)}$ (m, 3H)	1.32 - 0.65	3.14	1.32 - 0.65	8.41

SMS3 pH 5.0	week 0		week 9th	
**Signal**	**Chemical shift (ppm)**	**Integration value (normalized)**	**Chemical shift (ppm)**	**Integration value (normalized)**
$CH_{(j)}O$ (m, 1H)	5.38 - 5.11	1.00	5.38 - 5.11	1.00
$CH2_{(l)}O$ (m, 2H)	5.04 - 4.63	0.97	5.04 - 4.63	5.09
$CH2_{(c)}O$ (m, 2H)	4.49 - 3.76	2.92	4.49 - 3.76	21.54
$NCH2_{(d)}$ (m, 2H), $N(CH3)_{(2e)}$ (m, 6H)	3.18 - 2.22	13.53	3.18 - 2.22	51.68
$CH2_{(b)}$ (m, 2H)	2.22 - 1.68	2.43	2.22 - 1.68	11.84
$CH3_{(k)}$ (m, 3H)	1.68 - 1.37	3.58	1.68 - 1.37	9.99
$CH3_{(a)}$ (m, 3H)	1.37 - 0.69	4.31	1.37 - 0.69	35.78

Figure S6. ^{1}H NMR (600 MHz, $CDCl_3$) spectra of SMS3 with assigned chemical shifts and integration values of signals at pH 7.4 (A), and pH 5.0 (B).

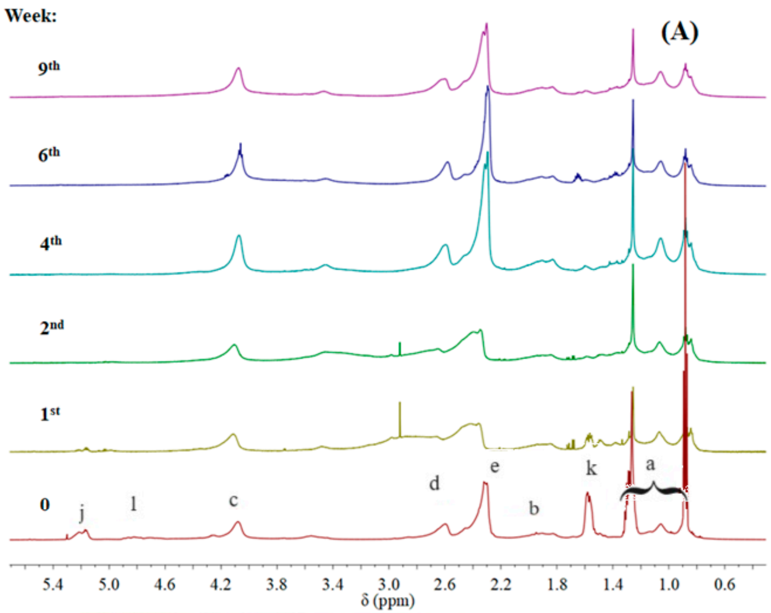

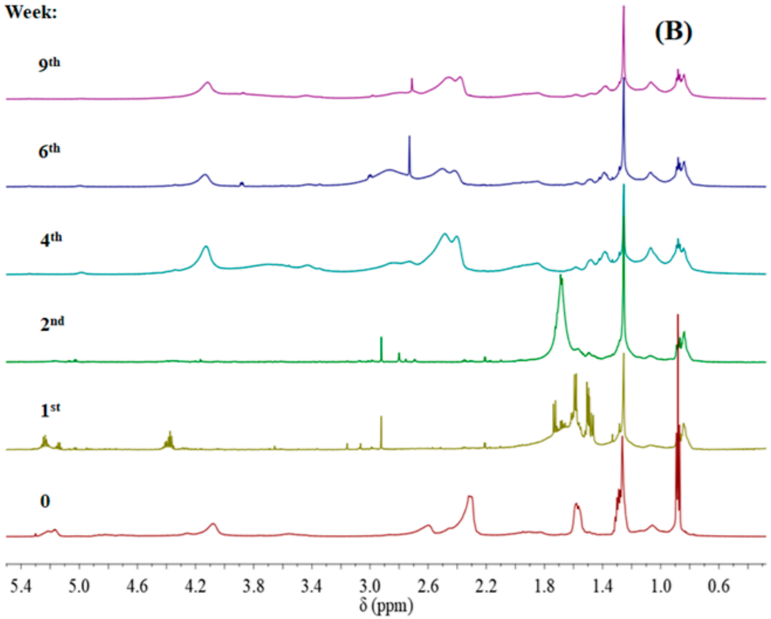

NMS3 pH 7.4	week 0		week 9th	
Signal	Chemical shift (ppm)	Integration value (normalized)	Chemical shift (ppm)	Integration value (normalized)
$CH_{(j)}O$ (m, 1H)	5.29 - 5.06	1.00	5.29 - 5.06	1.00
$CH2_{(l)}O$ (m, 2H)	4.91 - 4.64	0.59	4.91 - 4.64	1.81
$CH2_{(c)}O$ (m, 2H)	4.31 - 3.89	2.67	4.31 - 3.89	16.28
$NCH2_{(d)}$ (m, 2H), $N(CH3)_{(2e)}$ (m, 6H)	2.94 - 2.21	9.50	2.94 - 2.21	43.34
$CH2_{(b)}$ (m, 2H)	2.13 - 1.74	1.92	2.13 - 1.74	10.47
$CH3_{(k)}$ (m, 3H)	1.74 - 1.37	4.00	1.74 - 1.37	8.04
$CH3_{(a)}$ (m, 3H)	1.37 - 0.73	12.96	1.37 - 0.73	36.61

NMS3 pH 5.0	week 0		week 9th	
Signal	Chemical shift (ppm)	Integration value (normalized)	Chemical shift (ppm)	Integration value (normalized)
$CH_{(j)}O$ (m, 1H)	5.35 - 5.01	1.00	5.35 - 5.01	0.01
$CH2_{(l)}O$ (m, 2H)	4.89 - 4.56	0.61	4.89 - 4.56	0.02
$CH2_{(c)}O$ (m, 2H)	4.40 - 3.99	2.50	4.40 - 3.99	1.39
$NCH2_{(d)}$ (m, 2H), $N(CH3)_{(2e)}$ (m, 6H)	2.95 - 2.15	9.04	2.95 - 2.15	3.54
$CH2_{(b)}$ (m, 2H)	2.15 - 1.67	2.03	2.15 - 1.67	0.92
$CH3_{(k)}$ (m, 3H)	1.67 - 1.33	3.70	1.67 - 1.33	1.00
$CH3_{(a)}$ (m, 3H)	1.33 - 0.65	12.11	1.33 - 0.65	4.12

Figure S7. ^{1}H NMR (600 MHz, $CDCl_3$) spectra of NMS3 with assigned chemical shifts and integration values of signals at pH 7.4 (A), and pH 5.0 (B).

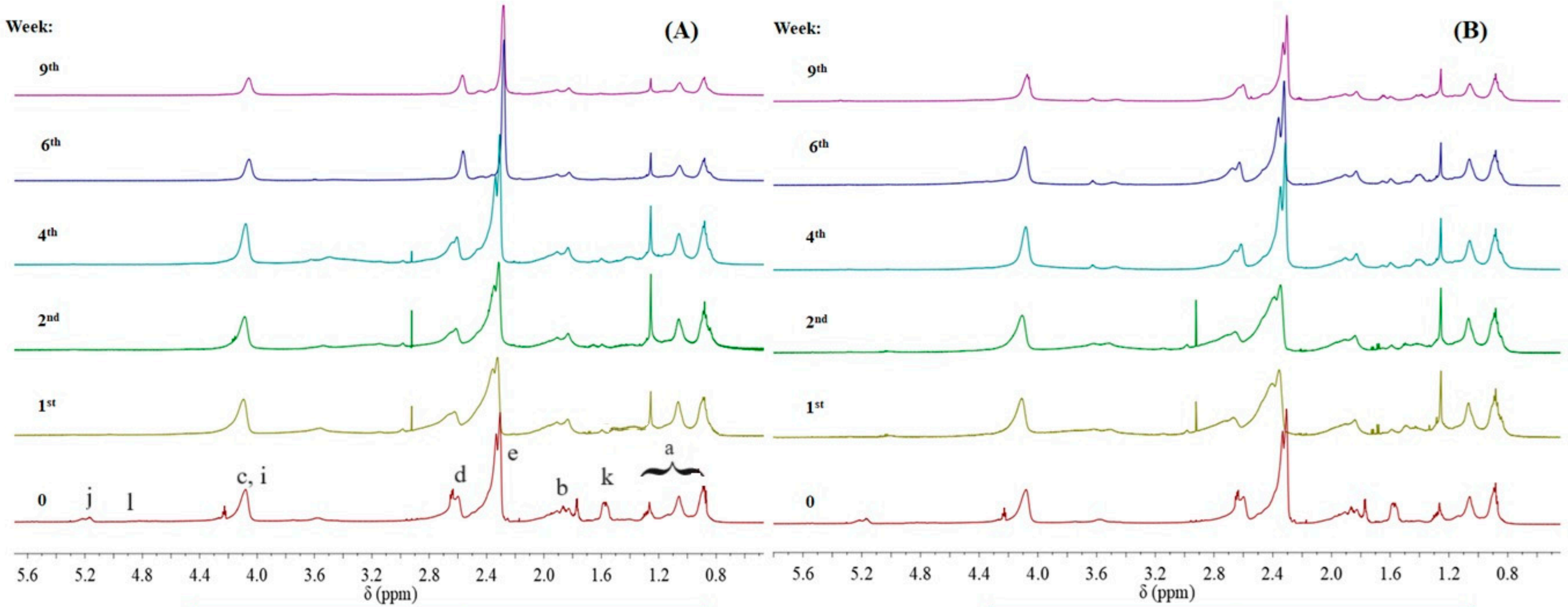

SMS4 pH 7.4	week 0		week 9th	
**Signal**	**Chemical shift (ppm)**	**Integration value (normalized)**	**Chemical shift (ppm)**	**Integration value (normalized)**
$CH_{(j)}O$ (m, 1H)	5.35 - 5.04	1.00	5.35 - 5.04	1.00
$CH2_{(l)}O$ (m, 2H)	4.93 - 4.65	0.75	4.93 - 4.65	0.84
$CH2_{(c)}O$ (m, 2H), $CH2_{(i)}O$ (t, 2H)	4.34 - 3.87	4.06	4.34 - 3.87	5.91
$NCH2_{(d)}$ (m, 2H), $N(CH3)_{(2e)}$ (m, 6H)	2.89 - 2.16	12.49	2.89 - 2.16	20.12
$CH2_{(b)}$ (m, 2H)	2.12 - 1.32	5.84	2.12 - 1.32	8.69
$CH3_{(k)}$ (m, 3H), $CH2_{(g)}$ (m, 4H), $CH2_{(h)}$ (m, 2H)	1.68 - 1.35	2.17	1.68 - 1.35	2.61
$CH3_{(a)}$ (m, 3H)	1.35 - 0.66	6.42	1.35 - 0.66	12.72

SMS4 pH 5.0	week 0		week 9th	
**Signal**	**Chemical shift (ppm)**	**Integration value (normalized)**	**Chemical shift (ppm)**	**Integration value (normalized)**
$CH_{(j)}O$ (m, 1H)	5.37 - 4.92	1.00	5.37 - 4.92	1.00
$CH2_{(l)}O$ (m, 2H)	4.91 - 4.61	0.64	4.91 - 4.61	0.70
$CH2_{(c)}O$ (m, 2H), $CH2_{(i)}O$ (t, 2H)	4.30 - 3.93	2.84	4.30 - 3.93	4.02
$NCH2_{(d)}$ (m, 2H), $N(CH3)_{(2e)}$ (m, 6H)	2.99 - 2.19	9.80	2.99 - 2.19	12.37
$CH2_{(b)}$ (m, 2H)	2.09 - 1.36	4.30	2.09 - 1.36	5.7
$CH3_{(k)}$ (m, 3H), $CH2_{(g)}$ (m, 4H), $CH2_{(h)}$ (m, 2H)	1.69 - 1.36	1.66	1.69 - 1.36	2.34
$CH3_{(a)}$ (m, 3H)	1.36 - 0.61	4.98	1.36 - 0.61	8.73

Figure S8. ^{1}H NMR (600 MHz, $CDCl_3$) spectra of SMS4 with assigned chemical shifts and integration values of signals at pH 7.4 (A), and pH 5.0 (B).

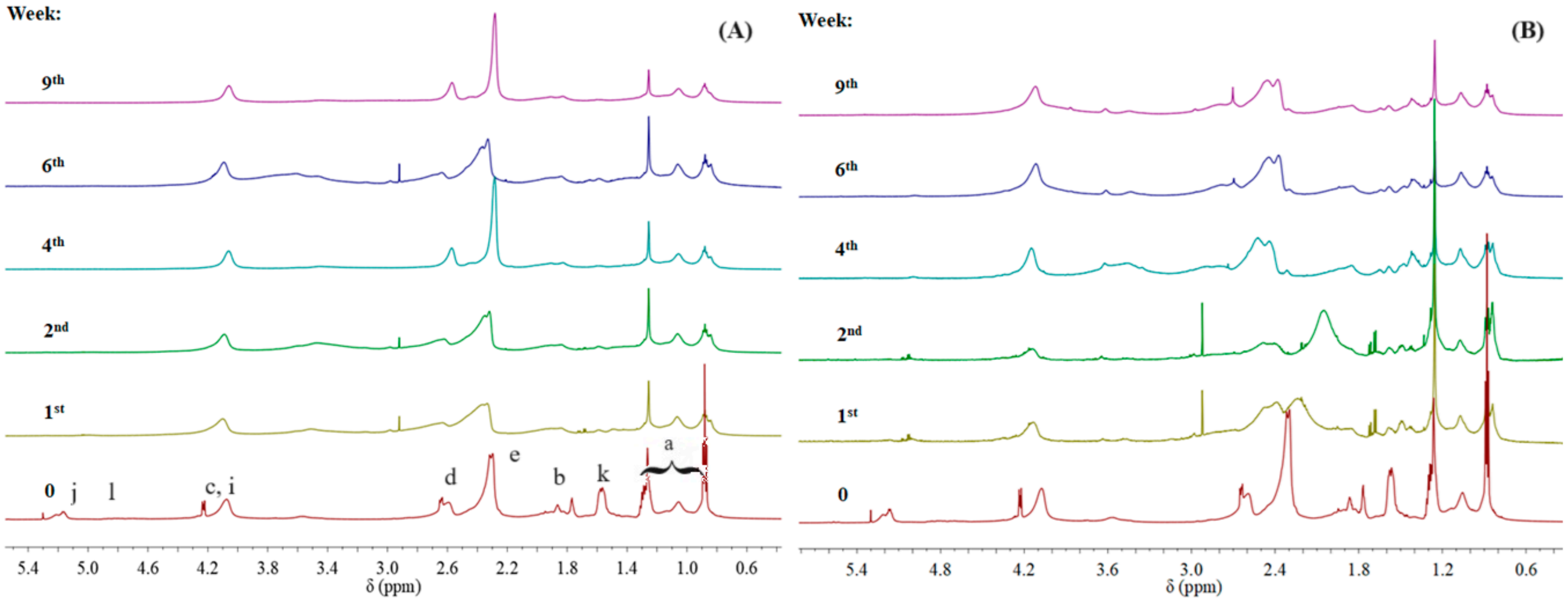

NMS4 pH 7.4	week 0		week 9th	
**Signal**	**Chemical shift (ppm)**	**Integration value (normalized)**	**Chemical shift (ppm)**	**Integration value (normalized)**
$CH_{(j)}O$ (m, 1H)	5.35 - 5.05	1.00	5.35 - 5.05	0.02
$CH2_{(l)}O$ (m, 2H)	4.91 - 4.61	0.39	4.91 - 4.61	0.01
$CH2_{(c)}O$ (m, 2H), $CH2_{(i)}O$ (t, 2H)	4.31 - 3.92	4.02	4.31 - 3.92	1.91
$NCH2_{(d)}$ (m, 2H), $N(CH3)_{(2e)}$ (m, 6H)	2.80 - 2.15	13.78	2.80 - 2.15	7.84
$CH2_{(b)}$ (m, 2H)	2.12 - 1.70	4.15	2.12 - 1.70	1.93
$CH3_{(k)}$ (m, 3H), $CH2_{(g)}$ (m, 4H), $CH2_{(h)}$ (m, 2H)	1.69 - 1.37	4.22	1.69 - 1.37	1.00
$CH3_{(a)}$ (m, 3H)	1.37 - 0.63	13.08	1.37 - 0.63	5.69

NMS4 pH 5.0	week 0		week 9th	
**Signal**	**Chemical shift (ppm)**	**Integration value (normalized)**	**Chemical shift (ppm)**	**Integration value (normalized)**
$CH_{(j)}O$ (m, 1H)	5.33 - 5.07	1.00	5.33 - 5.07	0.03
$CH2_{(l)}O$ (m, 2H)	4.92 - 4.56	0.44	4.92 - 4.56	0.10
$CH2_{(c)}O$ (m, 2H), $CH2_{(i)}O$ (t, 2H)	4.37 - 3.94	4.18	4.37 - 3.94	1.71
$NCH2_{(d)}$ (m, 2H), $N(CH3)_{(2e)}$ (m, 6H)	2.82 - 2.10	14.27	2.82 - 2.10	3.37
$CH2_{(b)}$ (m, 2H)	2.12 - 1.71	4.13	2.12 - 1.71	0.84
$CH3_{(k)}$ (m, 3H), $CH2_{(g)}$ (m, 4H), $CH2_{(h)}$ (m, 2H)	1.70 - 1.13	4.46	1.70 - 1.13	1.00
$CH3_{(a)}$ (m, 3H)	1.32 - 0.68	12.92	1.32 - 0.68	2.70

Figure S9. ^{1}H NMR (600 MHz, $CDCl_3$) spectra of NMS4 with assigned chemical shifts and integration values of signals at pH 7.4 (A), and pH 5.0 (B).

Table S3.Chemical shifts and integration values of signals assigned to SMS2 at pH 7.4, and pH 5.0.

SMS2 pH 7.4 i 5.0	week 0		week 9th	
**Signal**	**Chemical shift (ppm)**	**Integration value (normalized)**	**Chemical shift (ppm)**	**Integration value (normalized)**
C***H***$_{(j)}$O (m, 1H)	5.29 - 4.97	1.00	5.29 - 4.97	1.00
C***H2***$_{(c)}$O (m, 2H)	4.38 - 3.89	1.46	4.38 - 3.89	13.85
N***CH2***$_{(d)}$ (m, 2H), N(C***H3***)$_{(2e)}$ (m, 6H)	2.98 - 2.18	6.49	2.98 - 2.18	43.10
C***H2***$_{(b)}$ (m, 2H)	2.08 - 1.67	1.88	2.08 - 1.67	10.08
C***H3***$_{(k)}$ (m, 3H)	1.67 - 1.31	3.90	1.67 - 1.31	9.47
C***H3***$_{(a)}$ (m, 3H)	1.31 - 0.72	3.48	1.31 - 0.72	31.63

SMS2 pH 5.0	week 0		week 9th	
**Signal**	**Chemical shift (ppm)**	**Integration value (normalized)**	**Chemical shift (ppm)**	**Integration value (normalized)**
C***H***$_{(j)}$O (m, 1H)	5.31 - 4.95	1.00	5.31 - 4.95	1.00
C***H2***$_{(c)}$O (m, 2H)	4.29 - 3.95	1.26	4.29 - 3.95	2.93
N***CH2***$_{(d)}$ (m, 2H), N(C***H3***)$_{(2e)}$ (m, 6H)	2.64 - 2.13	4.83	2.64 - 2.13	6.90
C***H2***$_{(b)}$ (m, 2H)	2.07 - 1.76	1.39	2.07 - 1.76	2.26
C***H3***$_{(k)}$ (m, 3H)	1.75 - 1.32	3.30	1.75 - 1.32	4.32
C***H3***$_{(a)}$ (m, 3H)	1.32 - 0.71	3.09	1.32 - 0.71	7.21
